# Strain Tuning the Kinetics of Topotactic Phase Transitions in Epitaxial Oxide Films

**DOI:** 10.1002/smsc.70385

**Published:** 2026-09-02

**Authors:** Tadesse Billo Reta, Yan Li, Qingteng Zhang, Hyoungjeen Jeen, Irene Calvo Almazan, Gang Wan, Yongqi Dong, Huajun Liu, Sam Gruber, Guoxiang Hu, Eric M. Dufresne, Alec R. Sandy, Supratik Guha, Hua Zhou, Ho Nyung Lee, V. Starchenko, P. Ganesh, Dillon D. Fong

**Affiliations:** ^1^ Materials Science Division Argonne National Laboratory Lemont Illinois USA; ^2^ X‐ray Science Division Argonne National Laboratory Lemont Illinois USA; ^3^ Materials Science and Technology Division Oak Ridge National Laboratory Oak Ridge Tennessee USA; ^4^ Department of Physics Pusan National University Busan South Korea; ^5^ Condensed Matter Physics Department University of Zaragoza Zaragoza Spain; ^6^ Department of Mechanical Engineering Stanford University Stanford California USA; ^7^ Argonne National Laboratory Lemont Illinois USA; ^8^ Institute of Materials Research and Engineering Agency for Science Technology and Research Singapore Singapore; ^9^ The University of Tennessee Knoxville Tennessee USA; ^10^ Oak Ridge National Laboratory Oak Ridge Tennessee USA; ^11^ Georgia Institute of Technology Atlanta Georgia USA; ^12^ Pritzker School of Molecular Engineering University of Chicago Chicago Illinois USA; ^13^ Chemical Sciences Division Oak Ridge National Laboratory Oak Ridge Tennessee USA; ^14^ Center for Nanophase Materials Sciences Oak Ridge National Laboratory Oak Ridge Tennessee USA

**Keywords:** brownmillerite, epitaxy, materials science, nucleation, perovskite, phase transition, strain engineering, thermal diffusivity, thin film

## Abstract

We report the kinetics of the topotactic brownmillerite–perovskite phase transition in epitaxial strontium cobaltite (SrCoO_*x*_) thin films. Our findings reveal asymmetric switching kinetics between the oxygen‐deficient SrCoO_2.5_ and oxygen‐rich SrCoO_3_ phases, driven by the epitaxial strain state. Using phase‐field modeling, we demonstrate that nucleation and growth dynamics are governed by local oxygen concentration‐dependent diffusivity and surface oxygen flux, both of which are strain‐dependent. The perovskite phase exhibits higher strain tunability of these parameters compared to the brownmillerite phase, explaining the divergent kinetic pathways upon changes in epitaxial strain. Specifically, transitions on (LaAlO_3_)_0.3_(Sr_2_TaAlO_6_)_0.7_ (001) substrates are abrupt and nucleation‐dominated, characteristic of order–disorder transformations. Conversely, transitions on SrTiO_3_ (001) substrates are spatially uniform, with phase propagation originating from the film interface, characteristic of diffuse transformations. These results provide a framework for controlling topotactic transformation rates through strain engineering in functional oxides.

## Graphical abstract

We investigate the topotactic phase transition in epitaxial strontium cobaltite (SrCoO*_x_*) thin films. Our findings reveal asymmetric switching kinetics between the oxygen‐deficient SrCoO_2.5_ and oxygen‐rich SrCoO_3_ phases, driven by the epitaxial strain state. Using phase‐field modeling, we demonstrate that nucleation and growth dynamics are governed by local oxygen concentration‐dependent diffusivity and surface oxygen flux, both of which are strain‐dependent.

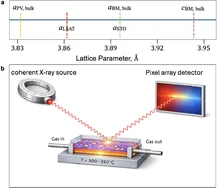

## 1 Introduction

It has long been recognized that point defects and oxygen vacancies in particular are critical to the functionality of many complex oxides, not only acting as electrical dopants but also as features essential for energy conversion and storage applications [1, 2, 3, 4, 5, 6, 7, 8]. In the ABO_3_ perovskites, of key importance is the nature of the B‐site; when the element can exhibit different oxidation states, the resulting concentration of oxygen vacancies can be large, leading to enhanced activity. One model system is La_1−*x*_Sr_*x*_CoO_3−*δ*_ (LSCO), a catalytically active oxide of interest for both the oxygen evolution and oxygen reduction reactions [9, 10, 11, 12, 13, 14, 15, 16, 17]. An applied potential allows for reversible tuning of the oxygen concentration, which has led to several electrochemical studies involving ionic liquids and gels: as the gate voltage becomes more positive, the loss of oxygen leads to a metal‐to‐insulator transition (and vice versa), as well as dramatic changes to the thermal, optical, and magnetic properties [18, 19, 20, 21, 22, 23, 24, 25].

The relative stability of Co^3+^ and Co^4+^ also allows reduction of the perovskite to the brownmillerite (ABO_2.5_) or square‐planar (ABO_2_) phases. In these phases, the oxygen vacancies become ordered, and the resulting change in crystal structure leads to the most significant changes in the material properties. This oxygen‐vacancy ordering has also recently attracted considerable interest in the nickelates, where superconductivity has been discovered and explored in Nd‐based oxides [26, 27, 28, 29]. In the nascent field of ionotronics [30, 31, 32, 33], various driving forces, including strain, can be used to vary the oxygen concentration, allowing for new device concepts based on the order‐disorder phase transition. Researchers are now designing vacancy‐driven systems employing sensors, artificial neural networks, and active learning for adaptive oxide electronics [34, 35, 36, 37].

Strontium cobaltite (SrCoO_*x*_ or SCO) is an end member of the LSCO series. The perovskite phase (PV‐SrCoO_3_) has a cubic structure and is a ferromagnetic metal, whereas the oxygen‐deficient brownmillerite phase (BM‐SrCoO_2.5_) has a vacancy‐ordered orthorhombic structure and is an antiferromagnetic insulator [18, 38, 39]. The SCO system is well known for its reversible topotactic phase transformation, in which oxygen can be extracted or inserted under relatively mild conditions. For example, annealing under low or high oxygen partial pressure converts the material between the BM and PV phases [18, 40, 41], and its defective surface can be active for a variety of other reactions [42, 43]. Much like LSCO, applying electrical pulses across the film can drive oxygen anions into or out of the lattice, thereby toggling the phase [44, 45, 46, 47, 48, 49]. Several groups have noted that such field‐driven topotactic switching makes SCO ideal for low‐power memristive devices [46, 50, 51]. For films epitaxially grown on single‐crystal substrates, stabilization of the cation sublattice can ensure that reduction of the perovskite is topotactic—there is no loss in crystallinity, although the strain state of the phase can change significantly. The oxygen vacancies in such films are ordered to form epitaxial superlattices composed of octahedral, tetrahedral, and square‐planar building blocks.

**FIGURE 1:**
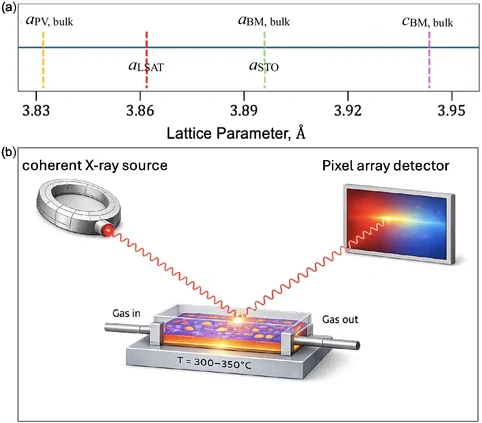
(a) Comparison of lattice parameters for bulk PV and BM phases relative to LSAT and STO substrates, illustrating epitaxial strain and lattice matching conditions relevant to the phase transition. (b) Schematic illustration of the coherent X‐ray scattering geometry and in situ experimental setup used to probe nucleation and topotactic phase transitions in epitaxial SrCoO_*x*_ thin films.

Variations in the chemical environment of SCO can then drive a phase transition, while the strain state imposed by an epitaxial substrate can shift the thermodynamic balance between phases [38, 52]. Figure 1a shows a comparison between the lattice parameters of different substrates and the bulk PV and BM phases. The in‐plane lattice parameter of the BM phase is very well matched to SrTiO_3_ (STO), favoring growth of SrCoO_2.5_ on SrTiO_3_ (001), as was illustrated by Jeen et al. [18]. In contrast, the lattice parameter of (LaAlO_3_)_0.3_(Sr_2_TaAlO_6_)_0.7_ (LSAT) lies midway between those of the BM and PV crystal structures (Table S1), and Jeen et al. showed that the PV phase is more easily grown on LSAT than on STO [18]. The kinetics of the transition can also be tuned with strain: this can occur through changes to the diffusivity, the rate of vacancy ordering, and the vacancy formation energy [42, 53, 54, 55], which control the concentration of vacancies prior to ordering. Tensile strain lowers the energy barrier for oxygen vacancy formation and can slow oxygen reincorporation [42, 54, 55], while compressive strain makes oxygen vacancies less favorable and can accelerate oxidation to the perovskite phase [3, 42, 56].

Here, we show using in situ X‐ray scattering (Figure 1b) and phase‐field modeling that the epitaxial strain in SCO/LSAT and SCO/STO heterostructures can be used to control the rate‐limiting step of the reversible PV  ↔︎  BM transition in SrCoO_*x*_. By directly linking strain to the kinetic pathway selection, this work clarifies how structural boundary conditions govern topotactic phase transitions in oxide thin films. These results establish a strain‐based design principle for controlling the transformation kinetics in complex oxide heterostructures.

## 2 Results and Discussion

Two types of heterostructures were prepared by pulsed laser deposition of ∼30 nm‐thick SrCoO_*x*_ films on both STO and LSAT (001) substrates [38, 52], leading to BM‐SCO and PV‐SCO, respectively. When the BM phase is heated in an oxygen environment, O_2_ dissociates at the surface, and the O^2−^ ions are subsequently incorporated within the vacant sites of the oxygen sublattice: this forms the nucleus of the PV phase that subsequently grows into the BM crystal. During PV reduction, O^2−^ ions diffuse to the surface to form O_2_, leaving vacancies within the oxygen sublattice; the BM phase only nucleates when the vacancies order to form the alternating octahedral/tetrahedral BM structure.

**FIGURE 2:**
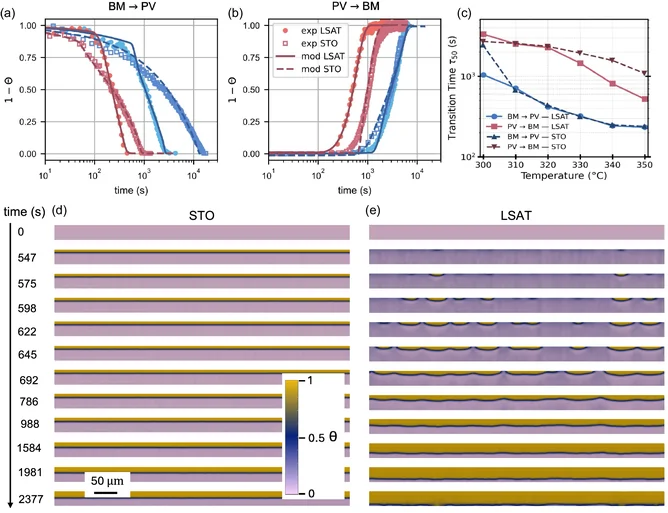
Comparison of phase‐transition kinetics and simulation dynamics in epitaxial SrCoO_*x*_ thin films on STO and LSAT substrates. (a) Experimental (symbols) and modeled (solid lines) normalized phase‐fraction evolution for the BM → PV transformation at both 300 °C (blue) and 350 °C (red) for SCO/LSAT (circles) and SCO/STO (open squares). Θ is a phase‐fraction (integral of *θ*) normalized by the initial and final composition, where *θ* = 0 is a BM and *θ* = 1 is a PV phase. (b) Corresponding evolution for the PV → BM transformation. Open squares represent STO substrates, and filled circles represent LSAT substrates. (c) Temperature dependence of the half‐transition time *τ*_50_ extracted from Johnson–Mehl–Avrami fits for both transformation directions on STO and LSAT. (d,e) Representative phase‐field simulation snapshots for the BM → PV transformation at 300 °C on STO (d) and LSAT (e). The images show slices perpendicular to the in‐plane direction and are arranged along a schematic time axis to illustrate the evolution of the phase boundary and the distinct propagation behavior on the two substrates.

The as‐grown films were first annealed in N_2_, resulting in 100% BM phase as evidenced by the single out‐of‐plane Bragg peak corresponding to the BM lattice parameter for both substrates [18]. Figure 2a,b presents the kinetics of the BM  →  PV and PV  →  BM transitions at both 300 °C (blue) and 350 °C (red) for SCO/LSAT (circles) and SCO/STO (open squares). The figures show the time evolution of the integrated intensity of the$00\frac{1}{2}$ Bragg reflection, a hallmark of the BM phase due to doubling of the unit cell size. A representative detector image and the stability of the selected ROI intensity prior to gas switching are shown in Figure S1. The intensity of the$00\frac{1}{2}$ peak confirms the initial presence of the BM phase in N_2_, where *θ* refers to the normalized local phase fraction with *θ* = 0 corresponding to the BM phase and *θ *= 1 corresponding to the PV phase (Figure 2a). Upon exposure to O_2_ at *t *= 0  s, the intensity decreases, indicating oxygen incorporation and subsequent transformation to the PV phase. Conversely, in Figure 2b, the absence of the$00\frac{1}{2}$ peak at the start (*θ *= 1) confirms the initial PV phase. After the introduction of N_2_ at *t *= 0  s, oxygen vacancies accumulate and order, leading to nucleation and growth of the BM phase, as illustrated by the increasing intensity of the$00\frac{1}{2}$ reflection.

There is a substantial difference in the kinetics of the transition for SCO/LSAT versus SCO/STO. On LSAT, the BM → PV transformations at both 300 and 350 °C exhibit an incubation period followed by rapid transformation and growth, whereas on STO the transformation proceeds gradually, consistent with bulk‐transport‐limited planar propagation through the film thickness. On the other hand, the PV → BM transformations in N_2_—reflecting the kinetics of vacancy ordering—are comparatively similar for both heterostructures. However, the transition remains faster for SCO/LSAT than for SCO/STO at 350 °C, despite the lower degree of epitaxial strain in the latter case.

Qualitatively different transitions observed in the same family of materials [57] at different driving forces (ionic gating under applied electric field) demonstrate a rich potential of such materials in tuning not only the static properties but also dynamics of the phase transition for a particular application. Our model treats the intrinsic phase kinetics associated with oxygen vacancy ordering/disordering under constant chemical potential at the surface of the film. Extending this framework to capture full transport phenomena would require the inclusion of charge kinetics and field‐driven ionic migration (electrophoretic effects), which remain absent from the current model but are likely essential for interpreting results such as those of Liang et al. [57].

To roughly estimate the kinetics of the phase transition, we determined *τ*_50_, i.e., the time required for the transition to reach 50% completion based on the normalized intensities shown in Figure 2a,b. The time parameter allows one to demonstrate the collective effects of temperature and substrate‐induced strain on the phase transition. Figure 2c shows the temperature dependence of *τ*_50_ for both the SCO/LSAT and SCO/STO heterostructures [52]. The BM → PV oxidation process (blue) exhibits a systematically decreasing *τ*_50_ with increasing temperature, in agreement with a thermally activated process. We further observe that the BM → PV transition for both substrates is faster than the inverse PV → BM transition at all temperatures. Assuming that all material parameters such as diffusivity are invariant with respect to the direction of the transition for both heterostructures, we conclude that these kinetic results are linked to the flux of oxygen ions into the surface during the BM → PV transformation and formation of ordered oxygen vacancies during PV → BM transformation. However, the similar transition times for BM to PV for both substrates (blue symbols in Figure 2c) do not necessarily indicate that the rate of vacancy disordering is strain independent, since *τ*_50_ represents intertwined processes, i.e., nucleation, growth, and the transport of oxygen. We have previously shown that the epitaxial strain also affects the initial oxygen‐vacancy concentration, with a higher oxygen‐vacancy concentration in BM‐SCO/STO than in BM‐SCO/LSAT. While this difference may contribute to the observed differences in the phase‐transition kinetics between SCO/LSAT and SCO/STO, we find that the kinetics are also strongly temperature dependent. In the phase‐field model, the contribution to the free energy from the elastic deformations due to lattice mismatch between the substrate and the film is an explicit term. Simulation results show that the differences in transition kinetics in the two heterostructures cannot be captured by changes to the free energy alone.

Further insight into the kinetics of the transition can be gained through analysis employing Johnson–Mehl–Avrami–Kolmogorov (JMAK) theory. The full set of experimental transition curves together with the corresponding JMAK fits for both substrates and transformation directions are provided in Figure S2. Although simple in form, the JMAK equation reflects the kinetics of the intertwined steps [58]:

(1) $$Y(t)=1-\exp\left[-kt^{n}\right]$$

where *Y*(*t*) represents the abundance of the new phase, *k* is the rate constant related to the transformation kinetics, and *n* is the Avrami exponent, which provides insight into the nucleation and growth mechanisms [59, 60]. The value of *n* represents the dimensionality of growth and the nature of nucleation (e.g., interface‐controlled vs. diffusion‐controlled), while *k* depends on temperature and different material properties. According to the JMAK theory, *k* is proportional to the rates of nucleation and growth of the new phase. We then estimate the characteristic time, *τ*_e_, where

(2) $$k\tau_{e}^{n}=1$$

**FIGURE 3:**
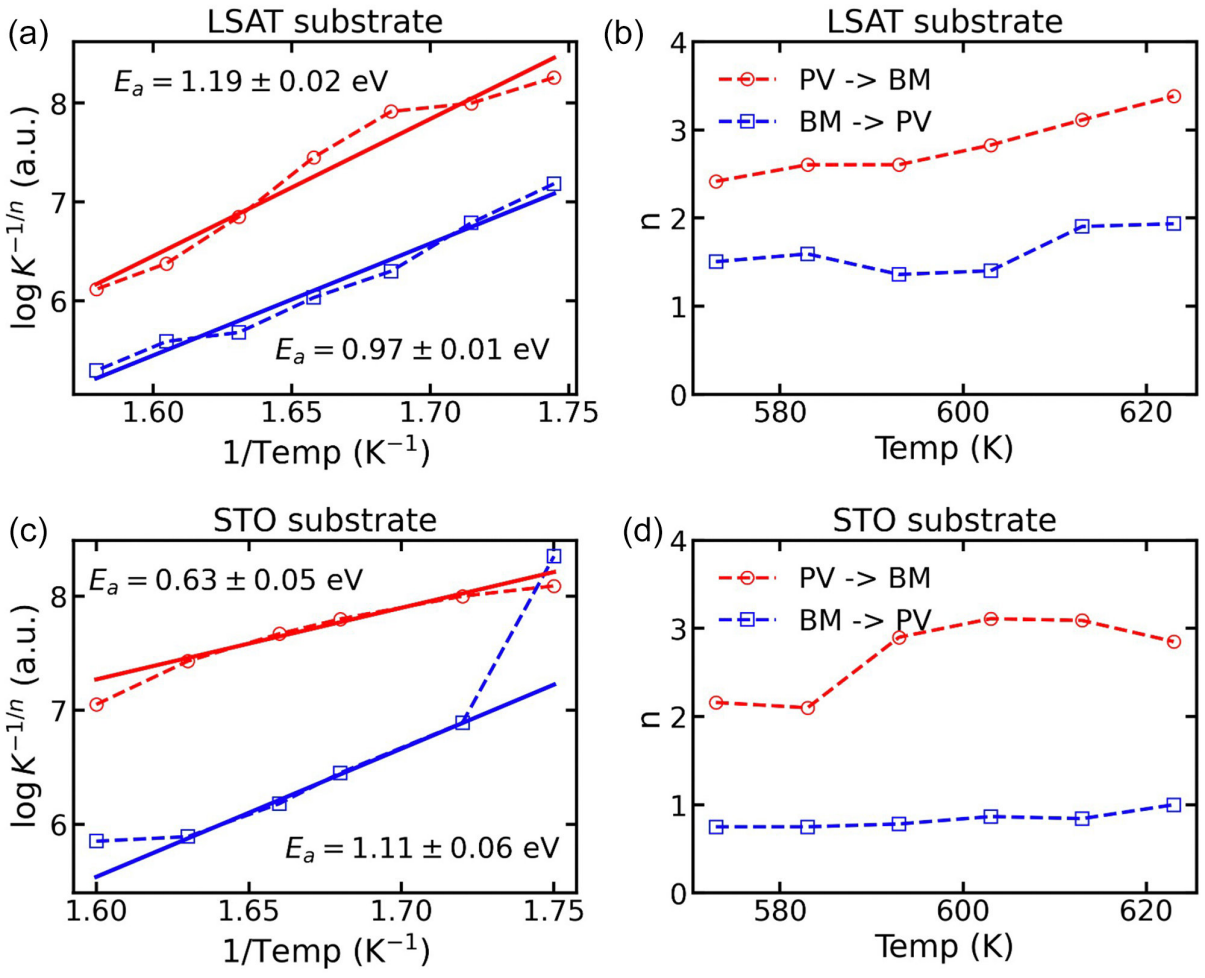
Temperature‐dependent kinetics of the topotactic phase transitions in epitaxial SrCoO_*x*_ thin films. (a) Arrhenius‐type dependence of the effective kinetic parameter log (*k* ^−1/*n*^) on inverse temperature (1/*T*) for films grown on LSAT. (b) Corresponding temperature dependence of the Avrami exponent *n* for LSAT. (c,d) Same analyses for films grown on STO substrates [52]. Symbols connected by dashed lines represent experimentally extracted values, while solid lines indicate linear fits used to estimate the effective activation energies. Red symbols correspond to the PV → BM transition, and blue symbols correspond to the BM → PV transition.

From the temperature dependence in Figure 2a,b **,** we can extract the activation energy for the kinetics of phase transition, *E*_a_, assuming$\tau_{e}\;\sim \;\exp\left(\frac{E_{a}}{k_{B}T}\right)$. The extracted activation energies, *E*_a_, based on linear fits to log (*τ*_*e*_), are shown in Figure 3a,c for SCO/LSAT and SCO/STO, respectively. The PV → BM transition on LSAT exhibits a higher activation energy of$E_{a}=1.19\pm 0.02$ compared to that for the STO substrate,$E_{a}=0.63\pm 0.05$ eV (red curves). The lower$E_{a}$ for SCO/STO (Figure 3c) is in agreement with DFT results [52], indicating a smaller activation barrier for vacancy hopping. Conversely, the activation energy for the BM → PV (blue curves) is lower for SCO/LSAT ($E_{a}=0.97\pm 0.01$eV) than for SCO/STO ($E_{a}=1.11\pm 0.02$ eV), suggesting that disorder may already exist within the oxygen sublattice of SCO/LSAT, facilitating conversion to the PV phase. The temperature dependence of the Avrami exponent, *n*, is shown in Figure 3b,d for SCO/LSAT and SCO/STO, respectively. The results indicate that the nucleation behavior for both BM formation and PV formation is higher dimensional for SCO/LSAT as compared to SCO/STO.

**FIGURE 4:**
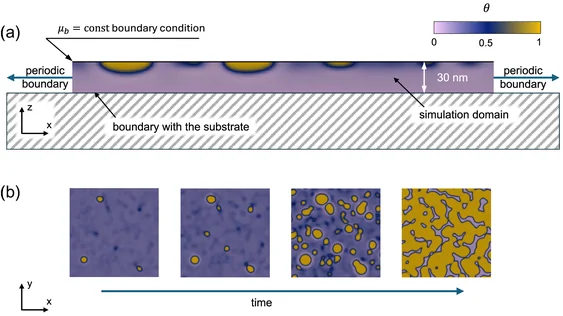
(a) Schematic representation of the simulation domain in the *XZ* plane. The film is represented as a simulation snapshot, where the color scale corresponds to the order parameter *θ*. (b) Top view of the simulation domain under a constant chemical‐potential boundary condition. This example demonstrates explicit nucleation events.

To better understand oxygen ion/vacancy ordering behavior during phase transition, we constructed a phase‐field model using the order parameter, *θ*, which reflects the local evolution of oxygen ions/vacancies governed by the Cahn–Hilliard equation [38]. The model explicitly couples the surface flux of oxygen ions/vacancies (implemented as a boundary chemical potential *μ*_b_) with bulk transport and ion/vacancy ordering with the goal of reproducing the time‐dependent transitions and determining the dominant rate‐controlling steps. A schematic representation of the modeled film is in Figure 4a, showing the propagation of oxygen ions and the perovskite phase (yellow) into the brownmillerite matrix (light purple).

The definition of the order parameter dictates that at *θ* = 0 the concentration of oxygen ions in the film corresponds to the BM phase, while *θ* = ‍1 indicates the PV phase. Figure 2a,b shows the time evolution of the order parameter overlaid on the experimental results. The simulations show that a lower driving chemical potential at the boundary, *μ*_b_, reproduces the early‐time plateau (surface‐exchange‐limited induction) for SCO/LSAT, while a higher *μ*_b_ is needed for SCO/STO to initiate BM → PV.

The modeling results further show that two distinct mechanisms are key to the BM → PV phase transition on both substrates. For SCO/STO, the process is bulk diffusion limited, and the growth of the new phase is a uniform plane propagating from the surface of the film to the bottom interface, as shown by the calculated images in (Figure 2d). For SCO/LSAT, the transformation occurs via slow saturation of the film with ions/vacancies until reaching the critical concentration for nucleation (Figure 2e). Overall, the propagation of the oxygen ion/vacancy front on both types of substrates proceeds from the top of the film to the bottom (Figures 2d,e and 4a). A top view of the simulated film (Figure 4b) demonstrates that nuclei start to form randomly across the film and eventually merge into a new phase.

Our simulation results suggest that on the STO substrate the rate‐limiting step is oxygen‐ion diffusion into the matrix (Figure 2d), whereas on LSAT the rate‐limiting step is the flux of oxygen ions through the surface of the film (Figure 2e). Therefore, a longer incubation time is observed, followed by a rapid phase transition on the LSAT substrate (Figure 2a). This behavior corresponds to the appearance of distinct phase nuclei on LSAT in contrast to the flat‐front propagation observed on STO. Diffusion‐limited processes have also been reported in other systems, such as La_1−*x*_Sr_*x*_CoO_3_ [57, 61]. In our study, using phase‐field modeling, we identify two distinct regimes—transport‐limited and reaction‐limited processes—which may also exist in La_1−*x*_Sr_*x*_CoO_3_ systems under different experimental conditions.

In previous work, we observed that the transition is correlated with the behavior of oxygen vacancies, and the dimensionality of the transition according to the JMAK model depends strongly on whether the phase is undergoing oxidation or reduction [52]. The dimensionality, *n*, can be used to interpret the type of interface dynamics and the ordering and disordering of oxygen vacancies during the phase transition. Adjusting the diffusion and interfacial flux parameters in the simulations reveals that ion incorporation during oxidation proceeds through a well‐defined nucleation process for SCO/LSAT, while for SCO/STO the lattice exhibits faster flux through the surface, leading to a more diffuse transition front (Figure 2d). Tables 1 and 2 show the values for the boundary chemical potential, *μ*_b_, and the diffusivity along the out‐of‐plane direction, *D*_33_. Positive values for *μ*_b_ drive the flux of the order parameter toward higher values (higher concentration of oxygen ions), whereas negative *μ*_b_ represents the flux of oxygen vacancies at the surface of the film. These correspond to the PV and BM values used in simulations to fit the experimental transition curves in Figure 2a,b. The unit timescale in the simulations is *t*_0 _= 1 s, and the unit length scale is *h*_0_ = 10 nm; therefore, the dimensionless diffusivity is$\hat{D}=D\frac{t_{0}}{h_{0}^{2}}$. Therefore, the reported values for *D*_33_ at 350 °C in Table 1 are on the order of 10^−18 ^m^2 ^s^−1^, and at 300 °C in Table 2 are on the order of 10^−19 ^m^2 ^s^−1^.

**TABLE 1:** Simulation dimensionless parameters that reproduce the experimental BM  →  PV and PV  →  BM phase‐transition kinetics on STO and LSAT substrates at 350 °C.

Substrate	BM → PV		PV → BM	
	LSAT	STO	LSAT	STO
${\hat{\mu }}_{b}$	0.32	0.58	−0.28	0.036
${\hat{D}}_{33}^{\mathrm{PV}}$	0.04	0.011	0.04	0.013
${\hat{D}}_{33}^{\mathrm{BM}}$	0.02	0.02	0.02	0.02

**TABLE 2:** Simulation dimensionless parameters that reproduce the experimental BM  →  PV and PV  →  BM phase‐transition kinetics on STO and LSAT substrates at 300 °C.

Substrate	BM → PV		PV → BM	
	LSAT	STO	LSAT	STO
${\hat{\mu }}_{b}$	0.45	3.0	−0.38	−0.07
${\hat{D}}_{33}^{\mathrm{PV}}$	0.004	0.00011	0.001	0.0011
${\hat{D}}_{33}^{\mathrm{BM}}$	0.002	0.0002	0.002	0.0025

Close analysis of the nucleation regime during the BM → PV transition on the LSAT substrate shows a lower flux of oxygen ions through the surface (i.e., a low value of the boundary chemical potential). The film is slowly saturated with oxygen ions, reaching the point at which nucleation occurs, arising from thermal fluctuations (Figure 2e for SCO/LSAT after 500–600 s). The transition then occurs rapidly. For SCO/STO (Figure 2d **)**, the value of the boundary chemical potential must be larger than that for SCO/LSAT (Table 1) to overcome the free‐energy slope introduced by the elastic part of the free energy in order to drive the BM → PV transformation.

Ab initio calculations show that epitaxial strain can alter oxygen vacancy migration barriers and correlation effects [55, 62]. This suggests that the ease with which oxygen can hop through the lattice (and thus the rate of phase change) is strain‐tunable. Calculations by Mayeshiba and Morgan also indicate that biaxial strain can significantly change the oxygen vacancy formation energy in several perovskites [55]. Experimentally, Petrie et al. [54] demonstrated that SrCoO_*x*_ films in biaxial tension contain a higher concentration of oxygen vacancies at ambient oxygen pressure, enabling greater catalytic activity for oxygen evolution.

The modeling results provide an estimate for the mobility of oxygen ions/vacancies for SCO/LSAT and SCO/STO (the diffusivity values in Tables 1, 2, and S2), highlighting the use of lattice strain as a lever for modulating oxygen sublattice chemistry—effectively tuning the material’s redox state and ionic conductivity without changing temperature or gas environment. The diffusivity of oxygen vacancies is given by

(3) $$D^{X}=g{\left(h^{X}\right)}^{2}\nu_{f}e^{-E_{a}/(k_{B}T)}$$

where *X* is either PV or BM, *g* is a geometric factor, *h* ^*X*^ is the hopping length, *E*_a_ is the activation energy associated with diffusion, and *ν*_f_ is the vacancy jump frequency. The two parameters key to the diffusivity in our experiments are activation energy and hopping length: the hopping length is proportional to the lattice parameter, and the activation energy is a scaling factor for the temperature dependence. Other material parameters can be found in Table S3. Based on our previous DFT calculations, the diffusion barrier for the oxygen ion along a 1D channel for BM on LSAT is 0.7 and 0.56 eV for BM on STO [52].

The simulation results suggest that the diffusion dependence on the local concentration is not trivial and must be treated as an anisotropic tensor for both the PV and BM phases. However, the most important factor is out‐of‐plane diffusivity, as the in‐plane diffusivity does not affect the transition curves significantly. Furthermore, the results in Figure 2a,b suggest that while strain affects the diffusivity of oxygen vacancies in the PV phase, the oxygen ion diffusivity in the BM phase remains constant for both SCO/LSAT and SCO/STO (Tables 1 and 2). Since the vacancy diffusivity is proportional to the hopping frequency (Equation (3)), which is not well defined in the SrCoO_*x*_ system, one can hypothesize that in SCO the increase in lattice parameter may decrease the probability of vacancy hopping, resulting in a reduced frequency. The simulations suggest that the out‐of‐plane diffusivity in the perovskite can change by a factor of four when switching from the LSAT to STO substrate, whereas it remains roughly constant for the brownmillerite phase.

## 3 Conclusions

In this study on transition kinetics in SrCoO_*x*_ films, a combination of experimental X‐ray scattering and phase‐field modeling reveals that the nucleation and growth of new phases depend on the local diffusivity of the species and the flux of oxygen ions/vacancies through the surface of the film, which in turn are dependent on the epitaxial strain imposed by the substrate. Phase‐field modeling suggests that strain on the LSAT substrate enhances oxidation by facilitating oxygen diffusion, leading to a faster BM → PV transition at all temperatures (Figure 2), whereas the STO substrate favors the BM phase by increasing the energy barrier for ion ordering and delaying the BM → PV transition. Simulations demonstrate that strain not only leads to an additional energy barrier for interfacial flux but also significantly affects the out‐of‐plane diffusivity for the PV phase while not affecting it strongly for the BM phase.

Integrating experimental observations with phase‐field modeling has emerged as a powerful approach for unraveling the coupled phenomena that occur during topotactic phase transitions. For instance, recent work by Zhang et al. [38] combined X‐ray experiments with phase‐field simulations informed by ab initio energetics to study defect dynamics in SrCoO_*x*_ heterostructures. This study revealed the intermittent fluctuations taking place between the BM and PV phases in a strained SrCoO_*x*_ film, with the phase‐field model attributing this behavior to the competition between elastic strain energy and defect ordering. Such integration between experiment and modeling provides greater understanding of how nanoscale processes such as oxygen‐vacancy ordering, domain nucleation, and phase growth give rise to the observed macroscopic switching behavior. This is the knowledge key to guiding the design of phase‐transition‐based devices from ionotronic memory elements to catalysts by illuminating how factors such as strain, electric field, and defect interactions can be engineered to achieve fast, reversible, and durable switching between functional oxide phases. Our results also underscore the importance of strain engineering not only in terms of tuning phase stability but also in controlling phase‐transition kinetics, which has significant implications for oxide‐based electronic and energy‐storage devices.

## Author Contributions

D.D.F., P.G., H.N.L., and V.S. conceived the idea and supervised the experiments, analysis, and modeling. H.J. and H.N.L. fabricated the samples. T.B.R., Y.L., Q.Z., I.C.A., G.W., Y.D., H.L., E.M.D., and H.Z. performed and participated in the synchrotron measurements and analysis. G.H., S.G., V.S., and P.G. performed the phase‐field modeling. A.R.S. and S. Guha provided critical suggestions. T.B.R., P.G., V.S., and D.D.F. co‐wrote the manuscript, and all authors commented on it.

## Funding

This study was supported by U.S. Department of Energy.

## Conflicts of Interest

The authors declare no conflicts of interest.

## Supporting information

Supplementary Material

## Data Availability

The data that support the findings of this study are available from the corresponding authors upon reasonable request.
