# Supplementary material for: Strain Tuning the Kinetics of Topotactic Phase Transitions in Epitaxial Oxide Films
Source: Small Sci. 2026 Sep 2;6(9):e70385. doi: 10.1002/smsc.70385 (PMC13573991; doi:10.1002/smsc.70385)
Supplement: Supplementary file 1 [file SMSC-6-e70385-s001.pdf]

**Supporting Information:**

# Strain Tunable Kinetic Pathways for Topotactic Phase Transitions in Complex Oxides

Tadesse Billo Reta[1], Yan Li[1], Qingteng Zhang[2], Hyoungjeen Jeen[3], Irene Calvo Almazan[1,9], Gang Wan[1,8], Yongqi Dong[2], Huajun Liu[1,7], Sam Gruber[11], Guoxiang Hu[4,10], Eric M. Dufresne[2], Alec R. Sandy[2], Supratik Guha[1,5], Hua Zhou[2], Ho Nyung Lee[3], V. Starchenko[6], P. Ganesh[4], and Dillon D. Fong[1]

[1]*Materials Science Division, Argonne National Laboratory, Lemont, IL 60439, USA*

[2]*X-ray Science Division, Argonne National Laboratory, Lemont, IL 60439, USA*

[3]*Materials Science and Technology Division, Oak Ridge National Laboratory, Oak Ridge, TN 37831, USA*

[4]*Center for Nanophase Materials, Oak Ridge National Laboratory, Oak Ridge, TN 37831, USA*

[5]*Pritzker School of Molecular Engineering, University of Chicago, Chicago, IL 60637, USA*

[6]*Chemical Sciences Division, Oak Ridge National Laboratory, Oak Ridge, TN 37831, USA*

[7]*Institute of Materials Research and Engineering, Agency for Science, Technology and Research, 2 Fusionopolis Way, Innovis #08-03, Singapore 138634, Singapore*

[8]*Department of Mechanical Engineering, Stanford University, Stanford, CA 94305, USA*

[9]*Condensed Matter Physics Department, University of Zaragoza, Zaragoza, Spain*

[10]*School of Materials Science and Engineering, Georgia Institute of Technology, Atlanta, GA, USA and*

[11]*Department of Mathematics, The University of Tennessee, Knoxville, TN 37996, USA*

## EXPERIMENTAL METHODS

Epitaxial $SrCoO_x$ thin films were grown on single-crystal substrates using pulsed laser deposition. Details of the synthesis procedure and structural characterization have been reported previously[1,2]. In the present work, the films were stabilized in the brownmillerite phase prior to measurements.

To study the reversible topotactic phase transition, the sample was mounted in a custom in situ reaction cell that allowed control of the gas environment while maintaining X-ray access. The temperature was regulated using a heater beneath the sample stage. Phase transitions between $SrCoO_{2.5}$ (BM) and $SrCoO_3$ (PV) were induced by switching the atmosphere between high-purity $N_2$ and $O_2$ at elevated temperatures.

In situ coherent X-ray scattering experiments were performed at beamline 8-ID-E of the Advanced Photon Source (APS), Argonne National Laboratory. Diffraction measurements were conducted near the $(001/2)$ Bragg reflection associated with the brownmillerite phase, enabling the evolution of the BM–PV phase transition to be monitored as a function of time. Additional details regarding the beamline configuration, coherence conditions, and data analysis procedures are provided in previous reports[1,2].

### Experimental protocol and data selection

The $SrCoO_x$ films were first stabilized at 400°C under flowing $N_2$ to ensure formation of the brownmillerite $SrCoO_{2.5}$ phase. The sample temperature was then reduced to the target value (300–360°C) and allowed to stabilize before switching the gas environment. After stabilization at the target temperature, the gas was switched to $O_2$ to induce the BM$\rightarrow$PV transition. After completion of the oxidation process, the atmosphere was switched back to $N_2$ to drive the reverse PV$\rightarrow$BM transition.

A representative detector image of the coherent X-ray scattering signal near the $(00\frac{1}{2})$ Bragg reflection is shown in **Fig. S1**(a). The red dashed circle indicates the region of interest (ROI) used for extracting the time-dependent intensity. **Fig. S1**(b) shows the normalized ROI intensity measured under $N_2$ prior to switching to $O_2$, confirming that the selected BM peak remains stable before the phase transition is initiated.

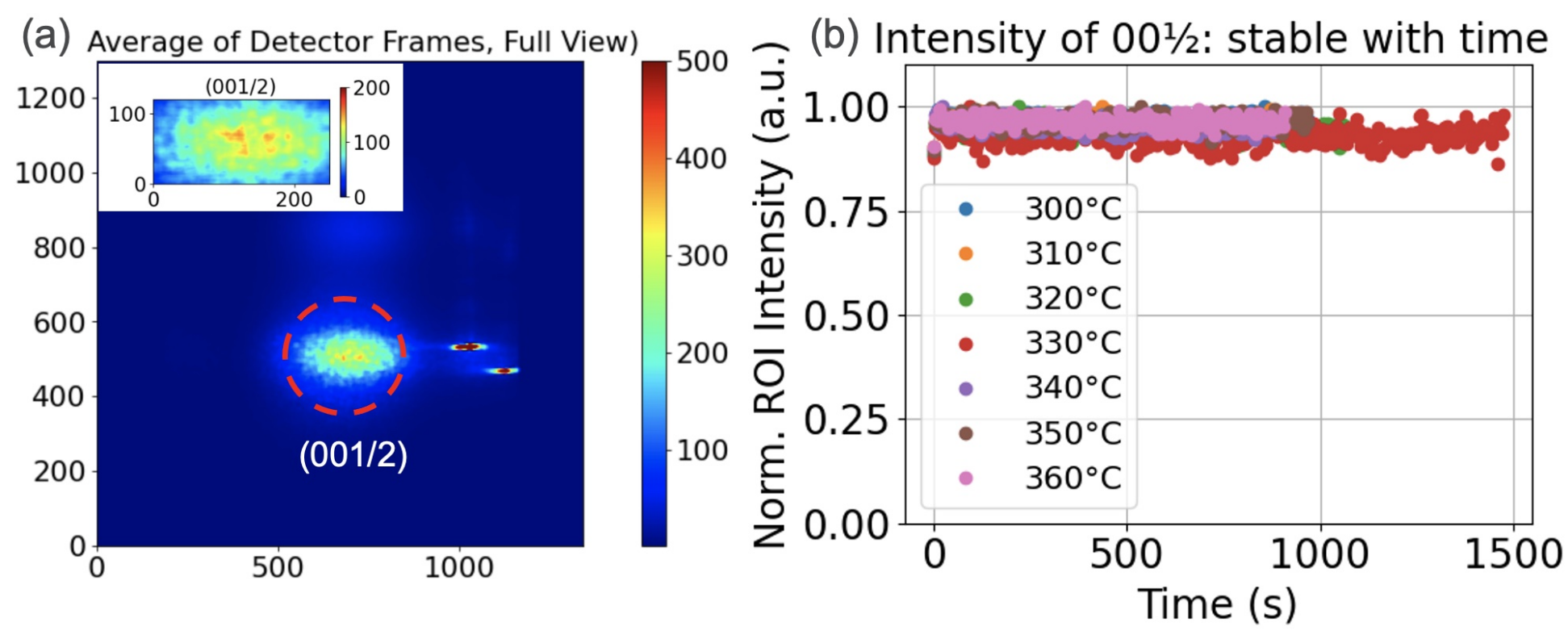


**Figure S1**: Detector view and ROI stability prior to gas switching. (**a**) Average detector image around the $(00\frac{1}{2})$ Bragg reflection measured during coherent X-ray scattering experiments. The red dashed circle marks the region of interest used for time-dependent intensity analysis. (**b**) Normalized ROI intensity as a function of time under $N_2$ at different temperatures, showing the stability of the BM-phase reflection before switching to $O_2$.

### Lattice parameters and strain estimates

The pseudocubic lattice parameters used in the strain analysis are summarized in **Table S1**. For the brownmillerite phase, the orthorhombic lattice parameters were converted to a pseudo-tetragonal representation for comparison with the cubic substrates.

$$a_{\mathrm{BM,pseudo}} = \frac{a_{\mathrm{BM},x} + a_{\mathrm{BM},y}}{2}$$

$$a_{\mathrm{BM,t}} = \frac{a_{\mathrm{BM,pseudo}}}{\sqrt{2}}$$

$$c_{\mathrm{BM,pseudo}} = \frac{a_{\mathrm{BM},z}}{4}$$

The in-plane mismatch was estimated using

$$\mathrm{mismatch} = \frac{a_{\mathrm{substrate}} - a_{\mathrm{film}}}{a_{\mathrm{substrate}}} \times 100\%.$$

These estimates show that STO is nearly lattice matched to the BM phase, whereas LSAT is closer to the PV phase. This difference in epitaxial boundary condition is consistent with the

substrate-dependent phase stability and transformation kinetics discussed in the main text.

**Table S1**: Pseudocubic lattice parameters and estimated in-plane mismatch values for $SrCoO_x$ phases on LSAT and STO.

| Substrate | Phase | Lattice parameter (Å) | Mismatch (%) |
|---|---|---|---|
| LSAT | Perovskite (PV) | 3.835 | +0.85 |
| STO | Perovskite (PV) | 3.835 | +1.79 |
| LSAT | Brownmillerite (BM) | 3.905 | -0.96 |
| STO | Brownmillerite (BM) | 3.905 | 0.00 |

## JMAK fitting and characteristic transition times

The time-dependent phase evolution was analyzed using the Johnson–Mehl–Avrami–Kolmogorov (JMAK) equation

$$Y(t) = 1 - \exp(-kt^n), \tag{1}$$

where $Y(t)$ is the transformed phase fraction, $k$ is the rate constant, and $n$ is the Avrami exponent.

The midpoint transition time $t_{50}$ was calculated from the condition $Y(t) = 0.5$

$$t_{50} = \left(\frac{\ln 2}{k}\right)^{1/n}. \tag{2}$$

The full set of experimental transition curves together with the corresponding JMAK fits for both substrates and both transition directions is shown in **Fig. S2**.

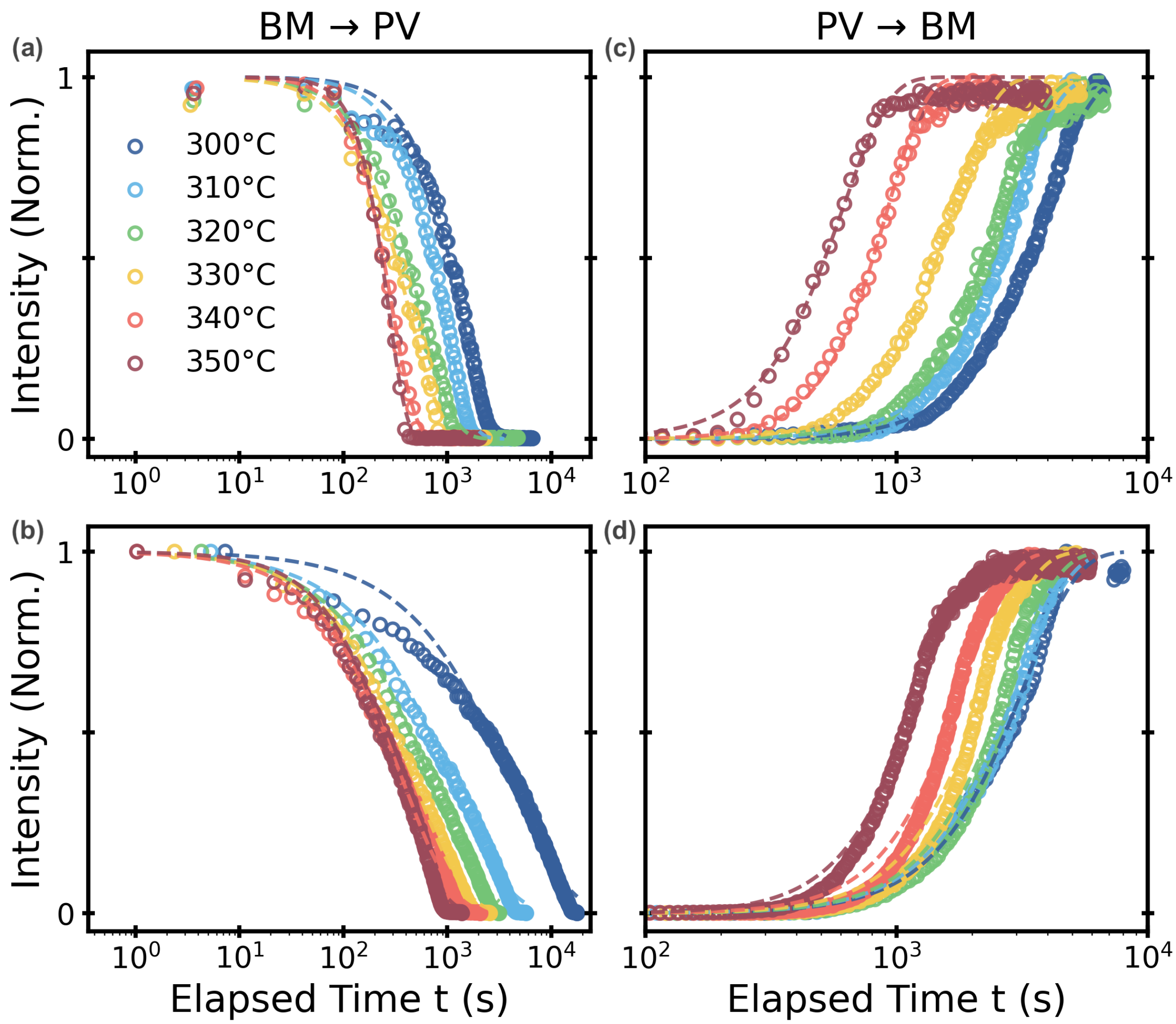


**Figure S2**: Full experimental transition curves and JMAK fits. (**a**,**b**) BM→PV transition kinetics for LSAT and STO substrates measured at 300–350°C. (**c**,**d**) PV→BM transition kinetics for LSAT and STO substrates over the same temperature range. Dashed curves show JMAK fits to the experimental data.

## SUPPLEMENTARY MODELING DETAILS

### Model overview

The present model extends our previous phase-field treatment[2] by incorporating elastic equilibrium and a constant chemical-potential boundary condition at the film surface to represent oxygen exchange with the gas environment. The model provides a qualitative description of the coupled

transport, ordering, and elastic effects governing the observed transition curves.

The order parameter $\theta$ describes the local oxygen content, with $\theta = 0$ corresponding to the BM phase and $\theta = 1$ to the PV phase.

### Diffusion tensor and effective mobility

Because the film inherits the in-plane lattice parameters from the substrate, we set

$$D_{11}^{\mathrm{PV}} = D_{22}^{\mathrm{PV}} = D_{11}^{\mathrm{BM}} = D_{22}^{\mathrm{BM}}$$

while the out-of-plane diffusivities $D_{33}^{\mathrm{PV}}$ and $D_{33}^{\mathrm{BM}}$ were adjusted independently to reproduce the experimental transition curves.

The effective diffusivity was represented as a harmonic mean

$$\frac{1}{D} = \frac{\theta}{D^{\mathrm{PV}}} + \frac{1-\theta}{D^{\mathrm{BM}}} \tag{3}$$

where $D^{\mathrm{PV}}$ and $D^{\mathrm{BM}}$ are the diffusivities associated with the perovskite and brownmillerite matrices, respectively.

To account for structural anisotropy, the diffusivities were written as diagonal tensors:

$$D^{\mathrm{BM}} = \begin{bmatrix} D_{11}^{\mathrm{BM}} & 0 & 0 \\ 0 & D_{22}^{\mathrm{BM}} & 0 \\ 0 & 0 & D_{33}^{\mathrm{BM}} \end{bmatrix}, \qquad D^{\mathrm{PV}} = \begin{bmatrix} D_{11}^{\mathrm{PV}} & 0 & 0 \\ 0 & D_{22}^{\mathrm{PV}} & 0 \\ 0 & 0 & D_{33}^{\mathrm{PV}} \end{bmatrix}. \tag{4}$$

The unit timescale in the simulations is $t_0 = 1$ s and the unit length scale is $h_0 = 10$ nm, so that the dimensionless diffusivity is defined as

$$\hat{D} = D\frac{t_0}{h_0^2}.$$

The in-plane diffusivities used in the simulations are listed in **Table S2**. These tests indicate that the in-plane diffusivity plays a comparatively minor role in determining the position and shape of the transition curves relative to the out-of-plane diffusivity.

**Table S2**: Dimensionless in-plane diffusivities used in the simulations.

| | BM→PV | | PV→BM | |
|---|---|---|---|---|
| **Substrate** | LSAT | STO | LSAT | STO |
| $\hat{D}_{11}(300°C)$ | 0.0001 | 0.00001 | 0.0001 | 0.0001 |
| $\hat{D}_{22}(300°C)$ | 0.0001 | 0.00001 | 0.0001 | 0.0001 |
| $\hat{D}_{11}(350°C)$ | 0.001 | 0.001 | 0.001 | 0.001 |
| $\hat{D}_{22}(350°C)$ | 0.001 | 0.001 | 0.001 | 0.001 |

**Elastic strain contribution**

The elastic free-energy density is written as

$$f_{\mathrm{el}}(\theta) = \frac{1}{2}\left[\boldsymbol{\varepsilon} - \boldsymbol{\varepsilon}^0(\theta)\right] : \boldsymbol{C}(\theta)\left[\boldsymbol{\varepsilon} - \boldsymbol{\varepsilon}^0(\theta)\right], \tag{5}$$

where $\boldsymbol{\varepsilon}$ is the total strain, $\boldsymbol{\varepsilon}^0(\theta)$ is the eigenstrain, and $\boldsymbol{C}(\theta)$ is the composition-dependent elastic stiffness tensor. We interpolate the elastic tensor as

$$\boldsymbol{C}(\theta) = \theta \boldsymbol{C}^{\mathrm{PV}} + (1-\theta)\boldsymbol{C}^{\mathrm{BM}}, \tag{6}$$

where $\boldsymbol{C}^{\mathrm{PV}}$ and $\boldsymbol{C}^{\mathrm{BM}}$ are the elastic tensors of the PV and BM phases.

Assuming Vegard-like behavior for the local lattice parameter,

$$a(\theta) = \theta a^{\mathrm{PV}} + (1-\theta)a^{\mathrm{BM}},$$

the eigenstrain is expressed relative to the substrate lattice parameter $a_0$ as

$$\varepsilon^0_{ij}(\theta) = \frac{a_0 - a(\theta)}{a(\theta)}\delta_{ij}, \tag{7}$$

where $\delta_{ij}$ is the Kronecker delta.

Mechanical equilibrium is imposed through

$$\nabla \cdot \boldsymbol{\sigma} = 0, \tag{8}$$

$$\boldsymbol{\sigma} \cdot \boldsymbol{n} = 0, \tag{9}$$

**Table S3**: Material parameters used in the simulations. The BM lattice parameters are listed in a pseudo-tetragonal framework for comparison with the substrates.

| **Lattice parameter** | | **Young's modulus** | | **Molar oxygen capacity** | |
|---|---|---|---|---|---|
| $a^{\mathrm{PV}}$ | 3.835 (Å) | $E^{\mathrm{PV}}$ | 174.38 (GPa) | $c^{\mathrm{PV}}$ | 0.088 (mol/cm$^3$) |
| $a_x^{\mathrm{BM}}$ | 3.905 (Å) | $E^{\mathrm{BM}}$ | 120 (GPa) | $c^{\mathrm{BM}}$ | 0.028 (mol/cm$^3$) |
| $a_y^{\mathrm{BM}}$ | 3.905 (Å) | **Poisson's ratio** | | | |
| $a_z^{\mathrm{BM}}$ | 3.936 (Å) | $\nu^{\mathrm{PV}}$ | 0.305 | | |
| $a^{\mathrm{LSAT}}$ | 3.868 (Å) | $\nu^{\mathrm{BM}}$ | 0.300 | | |
| $a^{\mathrm{STO}}$ | 3.905 (Å) | | | | |

where $\boldsymbol{\sigma}$ is the Cauchy stress tensor. The elastic deformations are small, so a linear elastic constitutive relation is used,

$$\boldsymbol{\sigma} = \boldsymbol{C}(\theta) : \left[\boldsymbol{\varepsilon} - \boldsymbol{\varepsilon}^0(\theta)\right].$$

Periodic boundary conditions are applied in the in-plane directions, and the displacement in the out-of-plane direction is fixed at the substrate interface.

The orthorhombic BM phase was compared to the cubic substrates using $a_x^{\mathrm{BM}}/\sqrt{2}$, $a_y^{\mathrm{BM}}/\sqrt{2}$, and $a_z^{\mathrm{BM}}/4$. In the present simulations we assume $a_x^{\mathrm{BM}} = a_y^{\mathrm{BM}}$, consistent with the idea that different in-plane BM orientations average the strain response in the film.

## Boundary condition for phase transformation

To represent the exchange of oxygen with the gas atmosphere, a Dirichlet boundary condition was imposed on the chemical potential at the film surface

$$\mu_b = \mathrm{const.}$$

Positive and negative values of $\mu_b$ correspond to oxidizing and reducing conditions, respectively.

## REFERENCES

[1]Q. Zhang, G. Hu, V. Starchenko, G. Wan, E. M. Dufresne, Y. Dong, H. Liu, H. Zhou, H. Jeen, K. Saritas, J. T. Krogel, F. A. Reboredo, H. N. Lee, A. R. Sandy, I. C. Almazan, P. Ganesh, and D. D. Fong, Phys. Rev. Lett. **129**, 235701 (2022).

[2]Q. Zhang, G. Wan, V. Starchenko, G. Hu, E. M. Dufresne, H. Zhou, H. Jeen, I. C. Almazan, Y. Dong, H. Liu, A. R. Sandy, G. E. Sterbinsky, H. N. Lee, P. Ganesh, and D. D. Fong, Advanced Materials **35**, 2305383 (2023).
